# Adaptive interventions to optimise the mobile phone-based smoking cessation support: study protocol for a sequential, multiple assignment, randomised trial (SMART)

**DOI:** 10.1186/s13063-022-06502-7

**Published:** 2022-08-18

**Authors:** Sheng Zhi Zhao, Xue Weng, Tzu Tsun Luk, Yongda Wu, Derek Yee Tak Cheung, William Ho Cheung Li, Henry Tong, Vienna Lai, Tai Hing Lam, Man Ping Wang

**Affiliations:** 1 School of Nursing, The University of Hong Kong, Hong Kong, 21 Sassoon Road, Pokfulam, Hong Kong; 2 Institute of Advanced Studies in Humanities and Social Sciences, Beijing Normal University, Zhuhai, China; 3 The Nethersole School of Nursing, Chinese University of Hong Kong, Ma Liu Shui, Hong Kong; 4 Hong Kong Council on Smoking and Health, Wan Chai, Hong Kong; 5 School of Public Health, The University of Hong Kong, Pokfulam, Hong Kong

**Keywords:** Adaptive trial, mHealth, Smoking cessation, Instant messaging, Stepped care

## Abstract

**Background:**

Mobile health (mHealth) is promising in developing personalised smoking cessation interventions. By using an adaptive trial design, we aim to evaluate the effectiveness of personalised mHealth intervention in increasing smoking cessation.

**Methods:**

This study is a two-arm, parallel, accessor-blinded Sequential Multiple-Assignment Randomised Trial (SMART) that randomises 1200 daily cigarette smokers from 70 community sites at two timepoints. In the first phase, participants receive brief cessation advice plus referral assistance to smoking cessation services and are randomly allocated to receive personalised instant messaging (PIM) or regular instant messaging (RIM). In the second phase, PIM participants who are non-responders (i.e. still smoking at 1 month) are randomised to receive either optional combined interventions (multi-media messages, nicotine replacement therapy sampling, financial incentive for active referral, phone counselling, and family/peer support group chat) or continued-PIM. Non-responders in the RIM group are randomised to receive PIM or continued-RIM. Participants who self-report quitting smoking for 7 days or longer at 1 month (responders) in both groups continue to receive the intervention assigned in phase 1. The primary outcomes are biochemical abstinence validated by exhaled carbon monoxide (< 4 ppm) and salivary cotinine (< 10 ng/ml) at 3 and 6 months from treatment initiation. Intention-to-treat analysis will be adopted.

**Discussion:**

This is the first study using a SMART design to evaluate the effect of adaptive mHealth intervention on abstinence in community-recruited daily smokers. If found effective, the proposed intervention will inform the development of adaptive smoking cessation treatment and benefits smokers non-responding to low-intensity mHealth support.

**Trial registration:**

ClinicalTrials.govNCT03992742. Registered on 20 June 2019.

**Supplementary Information:**

The online version contains supplementary material available at 10.1186/s13063-022-06502-7.

## Introduction

Evidence has shown that mobile phone-based interventions (mHealth) were effective in promoting abstinence by delivering remote, low-cost, scalable, and tailored cessation support [1–4]. Current mHealth studies mostly deliver automated and fix-scheduled text messages for cessation support [2, 3]. Mobile instant messaging (e.g. WhatsApp), as emerging alternatives of text messaging services, has the potential to incorporate more personalised behavioural and psychosocial support during the quitting process. Our formative qualitative interview of 21 community-recruited smokers showed the feasibility and acceptability of instant messaging as an mHealth platform for chat-based smoking cessation support [5]. Our large trial found chat-based cessation support combined with brief intervention effective in increasing smoking cessation service use and quitting [6]. Our pilot trial showed the feasibility and preliminary efficacy of chat-based behavioural support plus nicotine replacement therapy sampling [7].

However, the adherence of mHealth intervention is suboptimal [8]. Previous smoking cessation trials showed that only 17% and 24% of participants effectively engaged with the chat support [6] and mobile phone app use [9], respectively. Participants non-responding to the minimal mHealth behavioural support may require and could benefit from additional pharmacological or behavioural support [10]. Compared with delivering one-size-fits-all treatment regardless of participants’ responses, adaptively allocating interventions that are tailored to recipients’ responses can improve intervention outcomes while minimising unnecessary treatment burden [11, 12].

The adaptive approach is a scientific concept of stepped treatment intended to address patients’ unmet needs [13], which resembles the clinical practice of chronic disorder management such as depression, weight loss, and smoking cessation [14]. An assumption of adaptive interventions is that greater support (i.e. more intensive intervention) may lead to superior study outcomes and would be prescribed for all if resources are allowed [14]. Trials in reported use of adaptive design are increasing but mostly on drug development and dose-finding study [15]. Adaptive studies on smoking cessation are scant and no evidence-based guidelines for the clinical management of tobacco dependence treatment. The Sequential, Multiple Assignment, Randomised Trial (SMART) is an experimental design developed explicitly to optimise adaptive interventions and thus could be used to facilitate the development of more personalised mHealth cessation support [13].

In this study, the SMART design is proposed to optimise mHealth intervention in community-recruited smokers in terms of (1) altering the intensity of intervention to improve abstinence, (2) test the effectiveness of different adaptive mHealth strategies combined with evidence-based tobacco treatments, and (3) utilise self-determination to explore a more personalised smoking cessation treatment algorithm. Evidence showed that providing more choices of cessation services (e.g. nicotine replacement therapy, text messaging) increased quit attempts and long-term abstinence [16]. In this study, we provide optional combined cessation interventions based on smokers’ smoking status and preference. These interventions, including multimedia message [17, 18], nicotine replacement therapy sampling [19], active referral plus financial incentive [20], phone counselling [21], and social support [22], are evidence-based and supported by our previous trials. This study protocol describes the design and rationales of an adaptive trial that aims to evaluate the effectiveness of personalised mHealth intervention on quitting among community-recruited smokers.

## Methods

### Study design

**Fig. 1:**
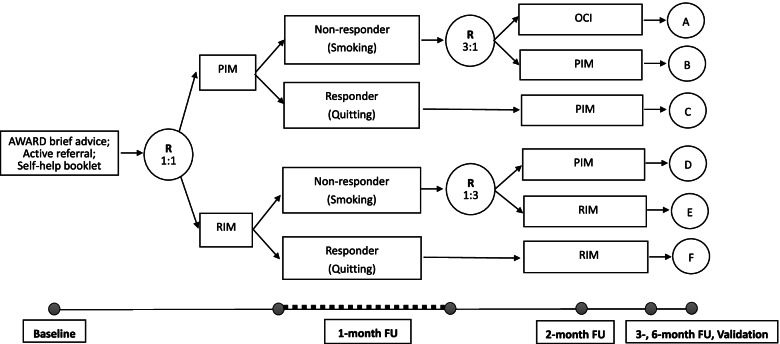
Study design overview. Abbreviations: R, randomisation; FU, follow-up; PIM, personalised instant messaging; RIM, regular instant messaging; OCI, optional combined intervention. Phase 1 intervention will be conducted during the first month after enrolment and phase 2 intervention will be conducted during the second and third months after enrolment

The SMART study is a two-arm, parallel, accessor-blinded randomised trial on daily smokers participating in the 10^th^ “Quit to Win” Smoke-free Community Campaign. The campaign is an annual smoking cessation contest organised by the Hong Kong Council on Smoking and Health to reach and motivate smokers to quit smoking [6, 23–26]. Participants are recruited in the community of all 18 districts in Hong Kong. The conducting and reporting of the proposed trial follow the Adaptive designs Consolidated Standards of Reporting Trials (CONSORT) Extension (ACE) guideline (Fig. 1) [27].

In the first phase (i.e. baseline), all eligible smokers receive brief cessation advice plus a referral to smoking cessation services. Participants are randomised with an equal probability to receive personalised instant messaging (PIM group) or regular instant messaging (RIM group). Evidence shows that early abstinence predicts long-term abstinence. In the second phase (i.e. 1-month follow-up), participants are assessed as responders (self-reported quitters) versus non-responders (continuing smokers, even a puff in the last 7 days) for the re-assignment of intervention. Non-responders in the PIM group are randomised to receive continued-PIM or additional optional combined interventions (OCI), in which they can choose their preferred interventions including (1) multi-media messages, (2) nicotine replacement therapy sampling, (3) financial incentive for active referral, (4) phone counselling, and (5) family/peer support group chat based on their preference, or a combination thereof. Non-responders in the RIM group are randomised to receive continued RIM or PIM. Responders in both groups continue to receive the initial intervention. All participants receive the 2-stage interventions for 3 months from baseline with 4 follow-ups at 1, 2, 3, and 6 months from treatment initiation.

### Participants and settings

Participants are recruited from 70 community sites (e.g. shopping malls and public areas) throughout all 18 districts in Hong Kong. Using the “foot-in-the-door” approach [28], trained smoking cessation counsellors proactively approach smokers, screen their eligibilities, invite them for participation, and deliver the baseline interventions once written consent is obtained. Smokers are informed that they will receive brief cessation advice and an exhaled carbon monoxide test, 3 months of instant messaging support, and sequent telephone follow-ups. Eligible participants are (1) Hong Kong residents aged ≥ 18 years, (2) daily smokers (smoked at least a cigarette per day in the past 3 months), verified by an exhaled carbon monoxide level ≥ 4 part per million (ppm), (3) able to communicate in Cantonese and read Chinese, (4) able to use instant messaging apps (e.g. WhatsApp, WeChat) on a mobile phone, and (5) motivated to quit or reduce smoking. Those who are having physical or cognitive difficulties in communication or currently participating in other smoking cessation programmes are excluded. Participants recruited using this method are largely similar to the smokers in the general population in terms of sex, age, and daily cigarette consumption [6].

### Randomisations and blinding

Within 3 days after baseline recruitment, participants are computer-randomised by an investigator who is not involved in recruitment. The first randomisation assigns participants into the PIM or RIM group with a random permuted block size of 2, 4, and 6 in 1:1 allocation ratio. At 1 month, a second randomisation is conducted among non-responders (smoke for even a puff in the past 7 days). In the PIM group, non-responders are further randomised to receive OCI or continue PIM with a block size of 4, 8, or 12 to ensure an allocation ratio of 3:1. Non-responders in the RIM group are randomised to receive PIM or continue RIM, with an allocation ratio of 1:3. The different allocation ratio in the second randomisation is designed to maximise the statistical power to compare OCI versus RIM, which is the co-primary comparison of the current trial (refer to 2.7). Smoking cessation counsellors, outcomes assessors, and statistical analysts are blinded to the group allocation.

### Interventions

All participants receive face-to-face brief interventions at baseline followed by 3 months of mHealth support via an instant messaging app. In phase 1 (i.e. the first month after enrolment), the PIM group receives personalised chat-based behavioural support and the RIM group receives generic, fix-scheduled messages on smoking cessation. We modify the subsequent treatments depending on the smoking status at 1-month follow-up and the second group allocation (Fig. 1 and Table 1).

**Table 1:** Adaptive intervention strategies embedded

	Adaptive intervention strategy			Type of strategy	Subgroup
	Phase 1 intervention	Tailoring variable assessed at 1 month (7-day PPA)	Phase 2 intervention		
1	PIM	Smoking	PIM+OCI	Adapt	A
2	PIM	Smoking	PIM	Maintain	B
3	PIM	Quitting	PIM	Maintain	C
4	RIM	Smoking	PIM	Adapt	D
5	RIM	Smoking	RIM	Maintain	E
6	RIM	Quitting	RIM	Maintain	F

Abbreviations: *PIM*, personalised instant messaging; *OCI*, optional combined intervention; *RIM*, regular instant messaging. Phase 1 intervention will be conducted during the first month after enrolment and phase 2 intervention will be conducted during the second and third months after enrolment

#### Baseline interventions

At baseline, all participants receive brief cessation advice plus a referral to a smoking cessation service [24]. Guided by the AWARD model, trained smoking cessation counsellors ask about participants’ smoking history (Ask), warn about the risks of smoking by the test results of exhaled carbon monoxide and a health warning leaflet (Warn), and advise participants to quit smoking within a pre-set quit date (Advise). With a separate consent signed, participants are encouraged to choose their preferred smoking cessation services using a pocket-sized referral card and share their contacts to the selected service providers. Service providers then contact participants within 2 weeks after enrolment (Refer). We boost the combined intervention during the period of mHealth support and at the 1- and 2-month follow-ups among those who fail to quit or relapse (Do-it-again) [6, 23, 24]. The face-to-face baseline intervention lasts for 5–10 min. All participants receive a 12-page self-help smoking cessation booklet.

#### Personalised instant messaging support (PIM)

There are two parts of the personalised behavioural support embedded in PIM intervention. First, a total of 24 fix-scheduled regular messages with contents including the harms of smoking, benefits and methods of quitting, dealing with craving, and information of smoking cessation services are sent to initiate the conversation. These regular messages are personalised to sex, age, daily cigarette consumption, exhaled carbon monoxide reading, intention to quit, and motivations of the participant. Those who intend to quit within 1 week or at the week of their pre-set quit date receive 5 messages/week, then cut down to 3 messages/week for the next 4 weeks and then 1 message/week for the last 7 weeks (Table 2). Second, smoking cessation counsellors perform real-time, interactive conversation with responded participants to provide personalised behavioural and psychological support. PIM intervention is effective to increase quitting in our previous trial [6]. Any cessation-related questions are answered as soon as possible within working hours (weekdays from 9:00 am to 6:00 pm).

**Table 2:** Overview of intervention frequency and time point

	Intention to quit	Allocation	Week 1	Week 2	Week 3	Week 4	Week 5	Week 6	Week 7	Week 8	Week 9	Week 10	Week 11	Week 12
**PIM regular message**	Within 7 days	Greeting	ooooo	ooo	ooo	ooo	ooo	o	o	o	o	o	o	o
	Within 30 days	Greeting	ooo	ooo	ooo	ooooo	ooo	o	o	o	o	o	o	o
	Within 60 days & Undecided	Greeting	o	o	o	o	ooo	ooo	ooo	ooooo	ooo	o	o	o
**Phase 2** **OCI (Subgroup A)**	MMM						×	×	×	×	×	×	×	×
	NRT-S						×							
	FIAR						×							
	PC						×^a^							
	SCG						\	\	\	\	\	\	\	\
**RIM**		Greeting	••	••	••	••	•	•	•	•	•	•	•	•
**Phase 2** **PIM (Subgroup D)**	Within 7 days and 30 days						ooooo	ooo	ooo	ooo	o	o	o	o
	Within 60 days and undecided						ooo	ooo	ooooo	ooo	o	o	o	o

Abbreviations: *PIM*, personalised instant messaging; *OCI*, optional combined intervention; *RIM*, regular instant messaging; *MMM*, multi-media messages; *NRT-S*, nicotine replacement therapy sampling; *FIAR*, financial incentive for active referral; *PC*, phone counselling; *SCG*, family/peer support group chat

^a^Schedule as required by participants

PIM intervention is delivered by smoking cessation counsellors well-trained in using transtheoretical behaviour change model (TTM) [29] and motivational interviewing (MI) [30]. The frequency and content of messages are guided by the TTM model and behavioural change techniques (BCTs) are used to maximise self-regulation and promote adjuvant activities for quitting [31]. MI counselling skills are used to increase their personal motivation in quitting. PIM intervention aims to engage and motivate participants to develop a personalised quit plan and facilitate further combined behavioural and pharmacological treatments for abstinence.

#### Optional combined interventions (OCI)

For non-responders in the PIM group randomised to receive OCI, smoking cessation counsellors explain and assist them to opt for sequent interventions at 1-month follow-up. Available intervention options include (1) multi-media messages, (2) nicotine replacement therapy sampling, (3) financial incentive for active referral, (4) phone counselling, and (5) family/peer support group chat. OCI participants who lost to follow-up or cannot make the decision at 1 month receive multi-media messages (default option) in addition to the PIM. Those who decline OCI intervention, if any, maintain the initial treatment.

##### Multi-media messages

Automated digital multi-media smoking cessation intervention increased self-efficacy in quitting and almost doubled the abstinence rate in a prior trial [18]. To increase entertainment and engagement, multi-media messages in the forms of smoking cessation-related pictures, web links, and short videos in combination with PIM are sent to OCI participants. Contents of the multi-media messages include thankful notes for joining the programme, tips for quitting, craving management, withdrawal symptoms, situation triggers, lapse and relapse, health information, benefits of quitting, enhancing motivations to quit, and introduction of smoking cessation service.

##### Nicotine replacement therapy sampling (NRT-S)

Previous RCTs found that NRT-S effectively increased quit attempts and abstinence [32, 33]. OCI participants who are willing to use medication assistance receive 1-week dosage of NRT-S (gum or patch) and used a card (Additional file 1: Appendix 1) containing instructions and potential side effects. Cessation counsellors confirm with participants for mailing address and contraindications to NRT use, including (1) recent (within 2 weeks) heart attack or severe arrhythmia, (2) unstable angina, or (3) pregnancy [34]. The dose of the NRT-S depends on participants’ daily cigarette consumption: 2 mg nicotine gum or 14 mg nicotine patch for those who smoke < 20 cigarettes per day and 21 mg nicotine patch for those who smoke ≥ 20 cigarettes per day. NRT use and potential side effects will be briefly explained based on a standardised script of the product instruction [35]. Cessation counsellors and research staff monitor side effects of NRT-S use through instant messaging interaction and telephone follow-ups. All adverse events will be reported to the IRB. Participants interested in a full course of NRT treatment will be referred to smoking cessation services.

##### Financial incentive for active referral

Our previous trial showed active referral plus a small monetary incentive was effective in increasing smoking cessation service use and abstinence [36]. To promote smoking cessation service use, participants who are verbally committed to using any smoking cessation services within 2 months will receive a pre-paid monetary incentive (HK$100, ≈ US$ 12.8) by post. A referral card (Additional file 2: Appendix 2) with the contact information of the nearest cessation clinic will be mailed along with the incentive. All treatment provided by the service providers are free of charge.

##### Phone counselling

An optional in-depth phone counselling is provided for OCI participants [21]. Counselling guidelines are MI-based, which was found more effective than standard telephone counselling (Additional file 3: Appendix 3) [21]. Trained cessation counsellors tailor the consultation to fit smokers’ motivational readiness, express understanding, and support their self-efficacy on quitting [30]. Counselling sessions last approximately 20 min and are audiotaped, of which 10% are randomly reviewed for quality check.

##### Family/peer-support chat group

OCI participants are encouraged to invite a family member or a close friend (peer) as their support partners to join an additional 2-month online group chat moderated by a cessation counsellor. Once obtained consent from the support partner via phone call or instant messaging, the cessation counsellor set up a chat group via instant messaging for personalised social support. The group discussion aims to engage the support partner to provide psychosocial supports and accompany participants to confront difficulties during the quitting process [37]. Cessation counsellors share additional information on quitting once weekly to initiate group discussion and provide real-time support as needed.

#### Regular instant messaging (RIM)

RIM group receives 16 regular messages with a tapering schedule from twice weekly in the first month to once a week in the following 2 months. These messages cover the same content as the regular messages in the PIM group. Additional 4 messages are delivered as reminders for participating in telephone follow-ups. RIM is unidirectional without real-time reply.

At 1-month, non-responders in the RIM group are further randomised to receive continued RIM or PIM (see the “Personalised instant messaging support (PIM)” section) for the following 2 months (phase 2 intervention).

#### Study fidelity

We recruit smoking cessation counsellors through university mass emails and advertising posters. All smoking cessation counsellors (university students and volunteers of non-governmental organisations) are required to attend a training workshop (6 h) on the study overview, proactive recruitment skills, and baseline intervention. We conduct a pre-test and post-test to evaluate their knowledge, attitude, and practice on smoking cessation.

On recruitment sites, at least one member of the research team oversees the recruitment progress and provides support to onsite counsellors as needed. All counsellors are instructed to follow a standardised recruitment script and an intervention checklist.

The instant messaging support and OCI interventions are supervised by a research nurse (SZZ) and a post-doctoral fellow (XW). The principal investigator, research staffs, and counsellors meet at least twice weekly to review trial conduct and discuss cases with side effects. All message dialogues are recorded and checked.

#### Define response vs. non-response

At the end of phase 1 intervention, all participants are assessed for intervention response. The procedure is embedded with 1-month follow-up. The 7-day point prevalence abstinence (PPA) is used to determine the response. A responder is defined as no smoking in the past 7 days whereas a non-responder is defined as smoking even a puff in the past 7 days. Participants who lost to follow-up at the 1-month are treated as non-responders.

### Data collection and follow-ups

Table 3 shows the schedule of data collection. At baseline, participants complete a questionnaire on their sociodemographic characteristics, smoking behaviour (daily cigarette consumption, the age of starting smoking, the time of having the first cigarette upon waking up in the morning, methods used in past quitting attempts), past quit attempts, intention to quit, smoking cessation service use, self-efficacy of quitting (perceived importance, difficulties and confidence of quitting measured on a Likert scale ranged 0–10), and nicotine dependence (Heaviness of Smoking Index, HSI) [38].

**Table 3:**
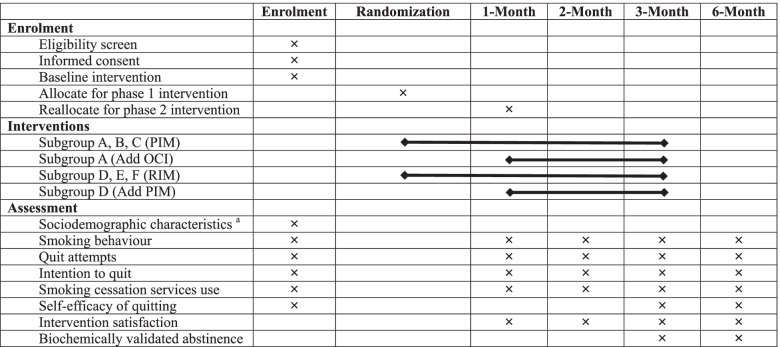
The schedule of enrolment, interventions, and assessments

^a^Sociodemographic characteristics include age, sex, education level, number of children, occupation, marital status, and household income

All participants are followed-up at 1, 2, 3, and 6 months to monitor the smoking and quitting behaviour, smoking cessation service use, and intervention engagement. Participants are considered lost to follow up after 7 missing calls and 1 voice message at different time and day of the week. Self-reported 7-day PPA at 3- and 6-month follow-up are invited for biochemical validation. Financial incentive of HK$500 (≈ US$64) will be given to both groups for passing the validation at 3 and 6 months (total HKD1000≈US$128) to increase the participation rate [20].

### Sample size and power calculation

G*Power is used to calculate the sample size [39]. The required sample size is based on the primary comparison of validated quit rate at 6 months (Fig. 1, Subgroup A+B+C versus Subgroup D+E+F). A meta-analysis showed the risk ratio of mHealth intervention on smoking cessation for biochemically validated abstinence at 6 months was 1.83 [40]. Assuming a 6-month biochemically validated quit rate of 5.0% in the in the D+E+F subgroups, 1200 participants will be needed (600 in each group) to achieve 80% power with a 5% false-positive error rate with an allocation ratio of 1:1.

In our previous mHealth trial, self-reported 7-day PPA at 1 month of the intervention and control group was 10.7% and 7.7%, respectively [6]. We estimate there will be 64 (= 600×10.7%) and 46 (= 600×7.7%) quitters for PIM and RIM groups at 1-month follow-up. Non-responders in the PIM (536, =600−64) and RIM groups (554, =600−46) will be reallocated by the second randomisation. Using an allocation ratio of 3:1 with a 5% false-positive error rate, a sample size of 402 (=536×3/4) in subgroup A and 416 (=554×3/4) in subgroup E has a power of 0.81 to test the co-primary outcome of validated quit rate at 6 months (Subgroup A versus Subgroup E).

### Outcomes

The primary outcome is biochemically validated abstinence at 6 months after treatment initiation using exhaled carbon monoxide (< 4 ppm) and salivary cotinine (< 10 ng/ml) [41]. Secondary outcomes include (1) biochemically validated abstinence at 3 months (end of treatment), (2) self-reported past 7 days abstinence, (3) smoking reduction by at least 50% of baseline consumption, (4) quit attempt (abstinence for ≥24 h), and (5) smoking cessation services use, defined by any use of provided treatments (e.g. counselling, medication, acupuncture).

#### Primary comparison

The main primary comparison is to between participants receiving PIM with additional OCI (Subgroup A+B+C) and participants receiving RIM with additional PIM (Subgroup D+E+F) (see Fig. 1). The co-primary comparison is PIM enhanced by OCI (Subgroup A) compare to RIM alone (Subgroup E) among non-responders to the phase 1 intervention. The hypothesis is that participants non-responding to the phase 1 mHealth interventions will benefit from additional personalised support.

#### Secondary comparisons

The main secondary comparisons are based on the hypothesis that more intensive cessation support will lead to increased abstinence:

1. The effect of the PIM enhanced by OCI (Subgroup A) versus PIM alone (Subgroup B) among non-responders to the phase 1 PIM treatment.
2. The effect of the RIM enhanced by PIM (Subgroup D) versus RIM alone (Subgroup E) among non-responders to the phase 1 RIM treatment.
3. The effect of PIM (Subgroup B+C) versus RIM (Subgroup E+F) among community-recruited smokers.

### Statistical analysis

Intention-to-treat analysis will be used with missing outcomes considered to have not changed from baseline [42]. We will compare the primary and secondary quitting outcomes of the primary comparison by using regression models with and without adjustment for important predictors of cessation outcomes, including previous quit attempts, intention to quit, and nicotine dependence [43]. Similar analysis will be used for co-primary and secondary comparisons, adjusting for multiplicity.

For sensitivity analysis, we will use multiple imputation by chained equation models to impute missing outcomes. Baseline characteristics predictive of abstinence will be included in the imputation model. Complete case analysis, where participants with missing abstinence outcomes are excluded, will be conducted.

For exploratory analysis, *t*-tests, supplemented by multivariable regression models, will be used to compare the baseline characteristics of responders and non-responders to the initial and subsequent treatments. Subgroup analysis by baseline characteristics on quitting outcomes will be conducted by interaction terms.

### Data management plan

The paper-based data will be scanned, stored on an external hard-disk, and locked in a cupboard with keys kept by the principal investigator. The Qualtrics will be used for data entry. Only the investigators and research staffs of the project will be permitted to access the raw data and/or study records. Data will be kept for 10 years or longer after the study is completed. Individual participants will not be directly identifiable from the dataset to be used for analysis.

### Post-trial qualitative evaluation

Qualitative evaluations using a subsample of participants receiving different intervention strategies will be conducted upon the completion of the 6-month follow-up. The semi-structured interview aims to explore participants’ experience and adherence of the intervention and obtain study feedback. Final sample size was determined by data saturation, which was until there was no new information arising from new interviews. A purposive sampling strategy was used to select a group of participants with varied sociodemographic characteristics (e.g. sex, age), smoking cessation outcomes, and intervention adherence. We anticipate that up to 20 participants will be included subject to data saturation. Additional written (for face-to-face interviews) or oral (for telephone interviews) informed consents will be obtained and all interviews will be audio-recorded and transcribed verbatim. The transcripts will be organised using a thematic framework based on topics specified in the interview guide and emerging themes identified through a process of familiarisation with the transcripts.

## Discussion

This is the first study using a SMART design conducted among community-recruited smokers to inform the delivery and effect of an adaptive mHealth intervention in quitting. The study design allows for multi-level comparisons of participants non-responding to the initial mHealth behavioural support and will inform the construction of adaptive interventions that balance quitting outcomes with resource consumption. mHealth instant messaging is the main gradient of this trial for both treatment groups. Firstly, we will test the effect of the personalised mHealth interaction as compared with regular messages on quitting. The findings may optimise the community-based cessation support and other behavioural change treatments utilising the low-cost and scalable mHealth strategy. Secondly, by re-allocating high-intensity treatment components to non-responders in initial treatment and as demonstrates need (optional interventions), the subsequent treatment provided personalised support while conserved treatment resources. Conventional tobacco treatments are additionally provided in options to encourage self-determination and autonomous motivation [44]. For responders of the phase 1 intervention, we will sustain the original intervention and adjust the content to prevent relapse. This may reduce treatment costs and burden as more intensive treatments are not necessary. This trial will operationalise and inform the development of personalised, adaptive, sequential mHealth treatment decisions made for smoking cessation in “real world” community settings.

This trial has several strengths. The recruitment is conducted in smoking hotspot that serves community-recruited smokers with less access to smoking cessation services and low intention to quit [24]. Proactive recruitment measures, where we take a “foot-in-the-door” approach to mobilise and motivate smokers in community settings, are adopted to recruit a more representative sample of common smokers. According to the TTM model, being ready to change within 1 month is at the preparation stage where outside support is influential to their change [29]. We adapt the frequency of mHealth support to the readiness to quit for a more personalised behavioural support. For non-responders, modifying treatment may be more effective earlier than later [45]. Our prior mHealth trial showed 87.3% (461/528) of non-responders and 69.8% (44/63) of responders at 1 month remained smoking and abstinence at 6 months, respectively [6]. These results supported the need for additional intervention for participants non-responding at the early stage thus to improve the long-term abstinence. For participants already responding to the initial intervention, the remaining initial treatment would be enough.

There are some potential limitations. Due to the nature of adaptive trials with multiple interventions, our study provides limited insight into the mechanisms and effects of each intervention component. Constrained by funding resources, we are not able to assess the long-term effects (e.g. 12 months post-treatment initiation). The quitting outcome at 3 and 6 months enables comparison with most current mHealth smoking cessation trials [1].

## Trial status

This is protocol version 1. Data collection of post-trial qualitative interviews is still in progress.

## Supplementary Information

**Additional file 1: Appendix 1.** Nicotine replacement therapy sampling use card.

**Additional file 2: Appendix 2.** Financial incentive for referral card.

**Additional file 3: Appendix 3.** Phone counselling guide.

**Additional file 4: Appendix 4.** Consent form.

## Data Availability

Not applicable.

## Abbreviations

AWARD: Ask, Warn, Advice, Refer and Do-it-again
ACE: Adaptive designs CONSORT Extension
BCTs: Behavioural change techniques
CONSORT: Consolidated Standards of Reporting Trials
COSH: Hong Kong Council on Smoking and Health
HSI: Heaviness of Smoking Index
IRB: Institutional Review Board
ITT: Intention-to-treat
mHealth: Mobile health
MI: Motivational interviewing
NRT-S: Nicotine replacement therapy sampling
OCI: Optional combined interventions
PPA: Point prevalence abstinence
ppm: Part per million
PIM: Personalised instant messaging
QTW: Quit to Win
RIM: Regular instant messaging
SMART: Sequential multiple-assignment randomised trial
TTM: Transtheoretical behaviour change model
