# Supplementary material for: Adaptive interventions to optimise the mobile phone-based smoking cessation support: study protocol for a sequential, multiple assignment, randomised trial (SMART)
Source: Trials. 2022 Aug 18;23:681. doi: 10.1186/s13063-022-06502-7 (PMC9387009; doi:10.1186/s13063-022-06502-7)
Supplement: Supplementary file 1 — Additional file 1: Appendix 1. Nicotine replacement therapy sampling use card. [file 13063_2022_6502_MOESM1_ESM.pdf]

香港吸煙與健康委員會
HONG KONG COUNCIL ON SMOKING AND HEALTH

HKU Med LKS Faculty of Medicine School of Nursing 香港大學護理學院

尼古丁補充劑-戒煙貼

戒煙貼______毫克　　　　每日使用時間___________

使用期間必須停止吸煙，
以免導致尼古丁吸收過量

尼古丁經皮膚吸收入體內，舒緩退癮症狀

用法：

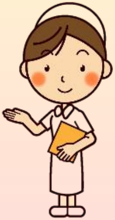

✓ 貼在清潔乾爽的皮膚上 (上臂、大腿等)
✓ 使用及更換後，請用清水洗手及避免接觸眼睛
✓ 定時更換；每天更換附貼位置
✓ 試用期間配合輔導員的跟進，效果更佳

X 附貼位置勿用潤膚露、藥膏或爽身粉
X 如影響睡眠則避免睡眠期間使用

Note: 此為一周劑量的試用品，如想接受完整尼古丁補充療法可與我地聯絡轉介去東華三院戒煙綜合中心接受免費治療。

*如有任何疑問或諮詢，請聯絡：
戒煙輔導員　電話：3917 6951

香港吸煙與健康委員會
HONG KONG COUNCIL ON SMOKING AND HEALTH

HKU Med LKS Faculty of Medicine
School of Nursing
香港大學護理學院

尼古丁補充劑-戒煙香口膠

約每___小時試用一粒　　　　每日最多使用________粒

使用期間必須停止吸煙，
以免導致尼古丁吸收過量

尼古丁經口腔吸收入血液中，舒緩退癮症狀

用法：

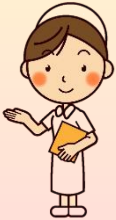

- ✓ 慢慢咀嚼10-15次，
- ✓ 將香口膠置於口腔壁與牙肉之間讓尼古丁吸收
- ✓ 重複以上步驟直至香口膠無味
- ✓ 試用期間配合輔導員的跟進，效果更佳

X 咀嚼時或使用前15分鐘避免飲食
X 不適合有牙齒疾患、口腔炎、喉炎、容易胃痛及配戴可拆除假牙的人士

Note: 此為一周劑量的試用品，如想接受完整尼古丁補充療法可與我地聯絡轉介去東華三院戒煙綜合中心接受免費治療。

*如有任何疑問或諮詢，請聯絡：
戒煙輔導員 電話：3917 6951
