# Supplementary material for: Adaptive interventions to optimise the mobile phone-based smoking cessation support: study protocol for a sequential, multiple assignment, randomised trial (SMART)
Source: Trials. 2022 Aug 18;23:681. doi: 10.1186/s13063-022-06502-7 (PMC9387009; doi:10.1186/s13063-022-06502-7)
Supplement: Supplementary file 2 — Additional file 2: Appendix 2. Financial incentive for referral card. [file 13063_2022_6502_MOESM2_ESM.pdf]

香港吸煙與健康委員會
HONG KONG COUNCIL ON SMOKING AND HEALTH

HKU Med LKS Faculty of Medicine
School of Nursing
香港大學護理學院

第十屆「戒煙大贏家」無煙社區計劃

親愛的 先生 / 小姐 :

感謝您參加第十屆「戒煙大贏家」!
隨函附上 **$ 100** 超級市場現金禮卷，鼓勵你參與**免費**嘅戒煙輔導。

您附近就腳嘅戒煙診所 不妨去睇下喇!
地址:

電話:

如果您成功戒煙，並應邀出席及通過核實測試，您可獲得 **$500** 現金獎。
如有任何問題，請聯絡戒煙輔導員(電話: 3917 6951) ， 或通過WhatsApp或WeChat與我們聯絡。

戒煙治療及研究組
香港大學護理學院

香港吸煙與健康委員會
HONG KONG COUNCIL ON SMOKING AND HEALTH

HKU Med LKS Faculty of Medicine
School of Nursing
香港大學護理學院

第十屆「戒煙大贏家」無煙社區計劃

親愛的　　　先生👨/小姐👱‍♀️:

🤗 感謝您參加第十屆「戒煙大贏家」🤗!
隨函附上 💰**$ 100** 💰超級市場現金禮卷，鼓勵你參與**免費**嘅戒煙輔導👩‍⚕️。

您附近就腳嘅戒煙診所⬇️不妨去睇下喇!
地址:

電話:

如果您成功戒煙，並應邀出席及通過核實測試，您可獲得 **$500** 現金獎。
如有任何問題，請聯絡戒煙輔導員(電話: 3917 6951) ， 或通過WhatsApp或WeChat與我們聯絡。

戒煙治療及研究組
香港大學護理學院🚭
