# Supplementary material for: Adaptive interventions to optimise the mobile phone-based smoking cessation support: study protocol for a sequential, multiple assignment, randomised trial (SMART)
Source: Trials. 2022 Aug 18;23:681. doi: 10.1186/s13063-022-06502-7 (PMC9387009; doi:10.1186/s13063-022-06502-7)
Supplement: Supplementary file 3 — Additional file 3: Appendix 3. Phone counselling guide. [file 13063_2022_6502_MOESM3_ESM.pdf]

第十屆「戒煙大贏家」無煙社區計劃

電話諮詢大綱（對象：OCI 組吸煙者）

日期	
時間	
輔導員名字	
參加者編號	
參加者名字	
參加者電話	

你好，我係香港大學護理學院戒煙大贏家嘅輔導員，好多謝你哩段時間對我地嘅支持！我地都睇到哩段時間你嘅努力，希望我地可以同你一齊加油，達到成功戒煙或減煙嘅目標。為保證我地嘅訪談質素同埋你嘅資料安全，今日我地嘅訪談會有電話錄音。訪談絕對安全，不會令閣下產生不安。全部資料都保密。訪談會佔用你大概 15-20 分鐘嘅時間，期間你隨時可以決定退出。多謝你嘅配合！

Themes	Questions	Follow questions	Conversation
（一）第一部分：了解吸煙習慣 （Engage）			
吸煙習慣<br>（預計時間：2 分鐘）	1 你宜家一日食幾多啊？<br>1#: ______	1.1 加入戒煙大贏家之前每日食幾多？	A. ***少咗***→問原因→問使用過嘅減煙方法<br>B. ***冇減到***→問原因→問現在是否想戒/減煙？
		1.2 加入戒煙大贏家之後有冇試過戒煙/減煙？	A. ***有***→問曾經使用的戒煙方法<br>B. ***冇***→問原因→之前有冇咩戒煙/減煙目標？
	2. 平時係咩情況下會食煙？（Triggers）	2#: ____________________<br>____________________	A.***有特定 trigger***→問原因→如果唔見到會唔會好 D？<br>B.***自己習慣，唔特別 trigger***→點樣開始食煙？
（二）第二部分：提供資訊 （Focus）			
請求許可分享資訊<br>（預計時間：2 分鐘）	3. 你平時有冇了解過吸煙嘅影響？	3.1 醫生有冇同你講過要戒煙啊？	A. ***有***→問點解→自己覺得有冇需要？<br>B. ***冇***→問 3.2
		3.2 家人或者身邊朋友有冇話希望你戒煙？	A. ***有***→問點解→自己點樣諗？有冇受影響？<br>B. ***冇***→咁自己有冇某一刻係覺得想戒？

	4. 有沒有因為吸煙引起的任何不適?	4.1 其實唔食幾日已經會有好多好處，會唔會想知多 D?	A. ***<u>有興趣知多 D</u>***→ 分享些簡單資訊 （***<u>Info.1</u>***）<br>B. ***<u>冇興趣</u>***→問點解→Affirm 理由
（三）第三部分：喚起動力 （Evoke）			
尋找原動力<br>（預計時間：2 分鐘）	5. 食煙對你來講有咩好處?	5#: ______________________<br>______________________	正向回應（Affirm）<br>接受及重複他的理由 (5#)
	6. 咁食煙對你來講有咩唔好處?	6.1 有冇健康方面嘅考慮?<br>6.1#: ______________________<br>______________________	A. ***<u>有</u>***→問疾病史 (心臟疾病，支氣管及肺部疾病等)<br>B. ***<u>冇</u>***→問有冇其他方面嘅考慮? （家人健康，二手煙及三手煙，煙貴，形象，吸煙地點減少等）
		6.2 你自己會唔會好擔心哩樣野?<br>6.2#: ______________________<br>______________________	A. ***<u>會</u>***→會點樣影響你嘅生活? /可唔可以俾個例子上一次係點樣令你擔心哩樣野?<br>B. ***<u>唔會</u>***→咁有冇其他食煙嘅唔好處?
		6.3 你覺得哩樣野會唔會令你想戒煙? /哩樣野會唔會係你想戒煙嘅動力?	A. ***<u>會</u>***→你之前戒煙會唔會諗起哩樣野?<br>B. ***<u>唔會</u>***→咁有冇諗過其他食煙嘅唔好處?<br>6.3# Other motivations: ______________________
戒煙重要性<br>（預計時間：1 分鐘）	7. 你覺得戒煙重要嗎?	7.1 如果 0-10 分，10 分係最重要。對你來講，戒煙係幾多分?<br><br>7.1#: ______________________<br>______________________	A. ***<u>6-10 分</u>***→都幾高！點解係 (7.1#) 咁高? （Downward question=change talk）<br>B.***<u>1-5 分</u>***→咁都有 D 重要喎~點解唔係 0?<br>C. ***<u>0 分</u>***→其實食煙對於我地身體嘅影響係比較耐先有明顯感覺嘅，你會唔會想了解多 D?
		7.2 有 D 咩情況會令你覺得戒煙對你來講更加重要?	7.2# : ______________________<br>______________________
戒煙信心<br>（預計時間：2 分鐘）	8. 對於戒煙，自己有冇信心?	8.1 有幾大信心可以成功戒煙? 0-10 分，10 分係最有信心。<br><br>8.1#: ______________________<br>______________________	A. ***<u>6-10 分</u>***→都幾高！點解係 (8.1#) 咁高? （Downward question=change talk）<br>B.***<u>1-5 分</u>***→點解係 (8.1#)? 點解唔係 0? 你覺得我地點樣做你嘅信心會再高 D?<br>C. ***<u>0 分</u>***→點解完全冇信心啊~?
		8.2 你估下幾多人靠自己戒煙會一次過成功?<br>8.2#: ______________________	其實得 3.5%嘅人靠自己直接唔食可以一次過成功嘅。但係超過 70%嘅吸煙者都好想戒煙，每年都有 40%嘅吸煙者努力戒緊煙。

		8.3 你覺得點樣可以再大 D 信心戒到煙？<br><br>8.3[#] : ____________________<br><br>____________________	A. ***有明確答案（eg.去戒煙診所）***→可以啊！ 哩種方法都係科學有效嘅，可以試下，我地宜家幫你轉介仲可以先俾$100 超市禮劵你喎 / 我地之前幫你轉介過，有冇去到？點樣？<br>B. ***冇明確答案***→如果你願意我地幫你一齊去努力戒煙，成功率都會高 D 嘅。有冇試過戒煙診所？我地宜家幫你轉介仲可以先俾$100 超市禮劵你喎！ 或者都可以寄 D 戒煙貼俾你試下先，覺得有效再去戒煙診所都 OK！
戒煙成功幾率<br>（預計時間：2 分鐘）	9. 你覺得戒煙成功幾率幾大？	9.1 0-10 分，10 分係一定會成功戒煙。<br><br>9.1[#]: ____________________	A. ***6-10 分***→都幾高！ 點解係 (9.1[#]) 咁高？ （Downward question=change talk）<br>B.***1-5 分***→點解係 (9.1[#])？ 點解唔係 0？<br>C. ***0 分***→點解完全冇信心啊~？
		9.2 如果我同你分享 D 其他人成功嘅方法會唔會再高 D？	A. ***有興趣知多 D***→ 分享些簡單資訊 （***Info.2***）→10.3<br>B. ***冇興趣***→問點解→Affirm 理由
		9.3 你聽完覺得點樣？有冇幫助？<br>9.3[#]: ____________________	
（四）第四部分：計劃 （Plan）			
總結需求<br>（預計時間：2 分鐘）	10. 咁其實你都好明白自己需要 D 咩野嘅！ 只不過可能仲未諗定點樣開始。	10.1 我地可以諗下係戒煙過程中有 D 咩壓力，再傾下點樣去一樣樣克服佢。	
		10.2 邊方面你覺得最有困難？<br><br>10.2#: ____________________<br><br>____________________<br><br>____________________	A. ***有明確答案（eg.身邊朋友都食）***→你覺得唔同佢地食煙會冇咁 Friend？ →其實關心你嘅人，會理解哩 D 係你個人嘅選擇。唔明白嘅人，都唔需要理會佢地嘅，始終我地咁樣做都係為咗自己，為咗關心自己嘅家人。<br>B. ***冇明確答案***→其實食或者唔食，都係你嘅選擇。如果你有諗過，就應該俾多個機會再努力去做！ 我地都會盡全力去幫你！
戒煙計劃<br>（預計時間：2 分鐘）	11. 我地啱啱討論咗關於戒煙嘅可能性喇。你都講咗戒煙對你係重要嘅，因為（7.1[#]討論的內容）______________. 你其實都有 D 信心可以戒到嘅，因為 （8.1[#]討論的內容）______________. 同埋戒煙都幾大可能性可以成功喇，因為（9.1[#]討論的內容）______________.		

	12. 現在你覺得你想點樣做？→其實我地啱啱都傾咗，如果我地（8.3# ____________） 同埋（9.3# ____________）可能都可以幫到你。
	13. 第一步你想點樣開始？不如我地一齊計劃下？→<br>（每日減 1-2 支/ 轉介[東華 or 博愛]+ $100 超市禮券[Address:____]/<br>NRT sampling[Address:____]/<br>朋友或家人群聊支持組[Tel No.__________________; Relationship:____________________] /直接唔食）
	14. 你想幾時開始行動？ 14#: ________________________
	15. 當你面對你最大嘅困難 （10.2#：___________________）, 你會點樣做？你可唔可以堅持？點樣可以幫到你？你可以諗下你最大嘅動力 （6#: _______________________________________）.

今次嘅電話輔導已經結束，好多謝你嘅參與，之後我地都會再有 WhatsApp 短信同電話跟進返你嘅戒煙情況。你有任何同戒煙有關嘅問題都可以係 WhatsApp 上同我地輔導員討論，希望你可以成功！ 加油！

電話諮詢完。

《輔導員自評》

你覺得被訪者回答自己成功戒煙時，語氣如何？

○1. 猶豫　○2. 敷衍　○3. 不開心、無奈　○4. 肯定　○5. 非常肯定　○6. 其他：__________

（整份電話跟進所用時間：_______分鐘）

Phone Counselling (Guided by Motivational Interviewing)

Aim:

By responding differently to smokers at different stages of change, counsellors will tailor the interventions to fit the smoker's motivational readiness. To assist smokers in moving from one stage to the next.

Skills:

1. **Avoid arguing**
   Gently diffuse client defensiveness. Confronting clients' denial can lead to drop out and relapse. When client demonstrates resistance to change, counselor changes strategies. (emphasizing personal choice and control can be helpful in this: "係呀，我地都明你可能都仲未唸定幾時開始戒嘅。你想點樣做都係你嘅選擇，完全係睇你自己點唸。").
2. **Express empathy**
   Be non-judgmental; listen reflectively; accept ambivalence; see the world through the client's eyes. Accurately understanding the client's experience can facilitate change. (let the patient know that he or she has been understood: "所以你屋企人不停同你講要戒煙令你果得好唔受尊重，你果得壓力好大所以食得更加多添係咪呀。").
3. **Develop discrepancy**
   Help client perceive difference between present behavior and desired lifestyle change. Clients are more motivated to change when they see what they're doing will not lead them to a future goal. (ie, between the patient's behaviour and personal values: "一方面你係想做一個好嘅家長，但係另一方面你有 D 擔心你個仔見到你食煙，可能唔係一個好嘅榜樣。對你來講平時如何去平衡？你點樣睇 ah？").
4. **Roll with resistance**
   Reframe client's thinking/statements; invite client to examine new perspectives; value client as being her own change agent. (ie, meet resistance with reflection: "所以你其實唔係好肯定你宜家需唔需要去改變你嘅食煙習慣。").
5. **Support self-efficacy**
   Provide hope; increase client's self-confidence in ability to change behavior; highlight other areas where client has been successful. (optimism that the patient is capable of making the change: "我見到其他人係同你食嘅完全一樣多，好快已經戒到。仲有好多食得仲多嘅人，都努力緊。").

Counselling tips：https://www.youtube.com/watch?v=1jfH055byg4

Interviewing model:

Brief Motivational Interviewing

Brief motivational interviewing can be integrated into any multi-session intensive tobacco treatment program. The elements of brief motivational interviewing involve FRAMES:

**F**eedback	Personalized information
**R**esponsibility	Freedom of choice; individual's responsibility for own health
**A**dvice	Need for change delivered clear, supportive, concerned manner
**M**enu	Strategies for change offered in a varied (menu) format
**E**mpathy	Empathetic, reflective, supportive style related to positive treatment outcomes
**S**elf-efficacy	Client's belief in ability to change is essential

Reflective Listening

The process of reflective listening involves hearing what the client says and either repeating or paraphrasing back to the client, or reflecting the feeling you believe is behind what the client says. Different levels of reflective listening can be distinguished

Working with resistance

To reduce client resistance, the counselor can use paraphrasing. Effective motivational interviewing involves a ratio of paraphrasing to questioning. The counselor should paraphrase two to three times as often as asking a question.

Information 1. 戒煙好處

身體即時變化:

1) 戒煙後二十分鐘血壓和脈搏會回到正常狀態，血液中的氧氣含量會增加回到正常；

2) 一天後罹患心臟病的危險性開始降低；

3) 三天後呼吸道狀況逐漸改善，肺活量也會恢復；

4) 兩週到三個月內，血液循環逐漸改善；

5) 戒煙五年後因肺癌造成的死亡率明顯降低，十年後肺癌的死亡率降低到和不吸煙的人幾乎完相同。

6) 其他各種癌症的發生率也都會減少。

生活改變:

1) 身體方面可以因為戒煙而降低罹患各種疾病，尤其是心臟血管疾病與癌症的威脅，使體能改善；

2) 心理上因為戒掉了吸煙的惡習，能自我控制，可增加自信心，而且不會再因為二手煙導致對別人的危害、污染環境等而有罪惡感;

3) 人際關係上，每天不必再耗費那麼多的時間吸煙，有更多時間與家人、親友等相處，也有更多的機會去做自己想做的事，不吸煙更可以成為兒女們的好榜樣；
4) 經濟上戒煙後省下不少錢來，更能節省因為吸煙導致生病所需的醫療費用。
【其實關於食煙對身體嘅壞處你可能已經了解好多，我地希望了解依 D 資訊唔係俾你壓力，令你邊食邊感覺罪惡，而係一個戒煙嘅原動力，係戒煙嘅過程中俾你力量。食煙永遠都只係一個選擇，你有得揀！】

Information 2. 其他人成功戒煙方法

Case 1：[阿偉]
戒煙原因：肺活量大大下降，感受到吸煙對健康的影響；尊重女朋友意見，女朋友曾表示如仍不戒煙便會分手
方法：東華三院提供尼古丁貼及尼古丁香口膠；接受青少年戒煙輔導（Youth quit line）；做運動舒發情緒及減壓
Case 2：[卓先生]
戒煙原因：女兒氣管過敏，從小常咳嗽，為了女兒和家人健康
方法：東華三院尼古丁補充劑；多喝水及運動來轉移注意力；醫生鼓勵增加 信心；跑步
【大部分人戒煙都係唸住靠自己意志力戒煙，係有人靠自己成功戒咗，但係有專業人士嘅幫助同支持會更加有效同輕鬆，如果你之前冇去到，我地都可以再幫你轉介去試多次】

Other information：

1. 吸煙/戒煙小知識

說法一: 戒煙沒有迫切性。

錯誤。其實由吸食第一口煙開始，已經對身體產生不良影響，吸煙愈多，不良影響愈大。雖然你不一定感覺到，但煙是隱形殺手，不要等到身患與吸煙有關的疾病時再戒煙。

說法二:間中吸一支煙都會對健康造成影響。

正確。吸煙會對健康造時即時影響，煙草在燃燒時會釋放出包括焦油、尼古丁及一氧化碳等有害物質的煙霧。這些煙霧含有 7,000 多種有害化學物質，如山埃同砒霜。當中超過 70 種物質更是致癌物，包括亞硝胺、乙醛、鉻等，這些毒素一旦進入身體，便會即時破壞各個器官，嚴重損害健康。

說法三: 吸煙的不良影響不會發生在青年或中年時期。

錯誤。吸煙對身心的不良影響包括短期及長期，有些害處很快會出現，例如中年便會心臟病發。愈早戒煙，好處就愈多。吸煙者如果在 60 歲時戒煙，可挽回因吸煙而減少的壽命多達 3 年，如果在 30 歲時戒煙，還可以賺回 10 年壽命。

說法四: 與吸煙有關的疾病有機會發生在自己身上。

正確。吸煙者往往認為不幸不會降臨在自己身上，這種想法是一種自我安慰的心理反應，藉此減少他們對吸煙而出現的擔憂，繼而導致他們對抗拒認清吸煙的壞處。其實每個人遇上不幸或幸運的機會都是平等的。

說法五: 電子煙對人體沒有傷害。

錯誤。香港政府對於電子煙的入口尚未有任何嚴謹監管，加上電子煙的致癌成分依然不明，不排除電子煙內的有害物質亦會令身體帶來不可估計的傷害。

說法六: 吸食電子煙不像普通煙會令人上癮。

錯誤。有些電子煙會含尼古丁，一樣會令人上癮。在香港販賣含尼古丁的電子煙屬違法。

說法七:長期吸煙的人在戒煙後，身體健康的好處都會即時呈現出來。

正確。有些長期吸煙的人聲稱對煙草早已適應，反而於戒煙後，潛伏在身體裡的暗病會即時發作，而這個說法完全沒有科學根據，每天繼續吸煙，對健康仍然有損害。

說法八:吸煙可令我提神，不吸反而精神差。

錯誤。由於煙草的成分，如尼古丁，可於七秒內上腦，因此驟覺能有提神效果，但效果十分短暫，而且危害健康。相反，想要提神可以採用其他方法。(例如: 如想即時提神，可以嘗試合口，只用鼻子快速吸氣及呼氣 15 秒，吸氣及呼氣的長度應該一致而平均，但愈短愈好。這樣可模擬我們運動時的呼吸頻率，故此有提神作用。)

說法九:戒煙有機會令體重暴增。

正確。由於戒煙後身體機能逐漸回復正常，味覺及嗅覺亦得到重新調整，胃口有可能因而大增。假如平日有不良的飲食習慣，體重便可能突然增加。所以只要有均衡飲食及做適量運動，便可以保持維持體態美，更可以享受美好佳餚。

說法十: 如果戒煙失敗，意味著我再沒有可能成功戒煙。

錯誤。你絕對可以戒除煙癮! 只要檢討上次戒煙過程的不足或障礙，避免犯下同樣錯誤，重新訂下戒煙計劃; 如有需要，更可同步配合戒煙輔導員的指引及使用戒煙輔助藥物，戒煙效果必定事半功倍。

2. 對抗煙癮小貼士

1）運用意志

認清自己的吸煙狀況後，作好準備；同時訂下戒煙日，承諾以後一口煙也 不吸，並貫徹始終。你更可告訴家人、朋友及同事你現正戒煙，以取得支持及 鼓勵。

2）拖延

當想購買或拿起煙時，盡量把腳步或動作放慢，或在心中慢慢地唸一至十， 趁這時回想自己戒煙的原因。如果你真的拿了捲煙出來，請先把它握在手中 並且不要點燃它，再重覆以上步驟。只要能順利度過那幾分鐘，你的煙癮就 可以退卻下來。 **3）分散注意力**

你可以洗個臉、聽喜歡的音樂或找人傾訴，讓自己冷靜下來，有助減壓。如 有需要，可閉目養神數分鐘或做些伸展運動。同時嘗試建立新的嗜好和興趣， 例如種花、看書、下棋、散步、做運動等，或參加社區中心舉辦的課程，以 有益身心的活動代替吸煙。

4）深呼吸、多喝水

千萬不要輕視深呼吸和喝水，這兩個簡單不過的方法同樣有助減退煙癮。多做 幾次緩慢的深呼吸運動，更可紓緩緊張情緒及重新提高集中力。

5）解手癮

因為你習慣拿煙的手勢，戒煙時難免有雙手空空的感覺。建議隨身攜帶減壓球 或原子筆，把玩時能轉移注意力，解決手癮問題。

3. 退癮症狀

退癮徵狀

在戒煙過程中，你會不時面對煙癮的挑戰，身體亦有可能因正在適應體內尼古丁減少而出現一些退癮徵狀。一般而言，退癮徵狀會出現兩至四周，不適感亦會隨時間逐漸減少。你可參考下列紓緩方式，及透過改善飲食習慣調整體質，以克服退癮徵狀帶來的不適。緊記保持均衡飲食，適量吸收不同的營養素，才能健康地成功戒煙！

退癮徵狀	原因	紓緩方式	飲食建議
焦慮、易怒、沮喪、情緒不穩、精神難集中	體內尼古丁減少，產生生理變化	• 暫時離開有壓迫感的地方<br>• 轉移注意力，將工作或活動分段完成<br>• 將全身肌肉繃緊後再完全放鬆，配合深呼吸，重複交替數次<br>• 洗溫水澡、散步、找朋友聊天	• 補充能紓緩壓力的維他命A、B及C，例如番薯、果仁、金奇異果<br>• 進食前及進食時飲水，提高飽肚感及填補無法吸煙的失落感<br>• 建議每日喝最少2000毫升或約八杯白開水
難以入睡	體內尼古丁減少，令睡眠習慣改變	• 做些緩和運動再入睡	• 避免飲用含咖啡因或酒精的飲品
便秘	體內尼古丁減少，令腸道蠕動改變	• 保持輕鬆心情<br>• 視乎情況使用溫和通便劑	• 多吃水果及富膳食纖維的高纖飲食，包括全穀類、豆類、西蘭花、香蕉等，可幫助排便
咳嗽、口乾	肺部正在清除焦油和黏液	• 多喝溫水<br>• 不要吃生冷食物，注意保暖，以免刺激引起咳嗽	
食慾增加	煙草中的尼古丁會降低食慾，加上戒煙後味覺和嗅覺改善，所以會增加饑餓感	• 選擇低熱量食物，進餐時先喝清湯，吃青菜，最後才吃肉類和主食，細嚼更有飽足感，不致於戒煙期間攝取過多的熱量和脂肪<br>• 咀嚼低卡路里的小吃，如高纖乾果、高纖燕麥條、小甘荀、無糖香口珠、薄荷糖或其他生果等	

4. 常見問題

Q: 間中吸一支煙是否不會對健康造成影響?

A: 吸煙會對健康造成即時的影響，煙草在燃燒時會釋出包含焦油、 尼 古 丁 和 一 氧 化 碳 等 有 害 物 質 的 煙 霧。 這 些 煙 霧 含 有 7,000 多 種 有害化學物質，如山埃和砒霜。當中超過 70 種物質更是致癌物，包 括亞硝胺、乙醛、鉻等，這些毒素一旦進入身體，就會即時破壞各個 器官，嚴重損害健康。吸煙不但危害個人健康，二手煙亦影響非吸煙 人士的健康，所以切勿嘗試第一口煙。

Q ： 應該在何時戒煙?

A ： 儘早戒煙，好處就越多，吸煙人士在 60 歲時戒煙，可挽回因吸煙而減 少的壽命達三年之多。如果在 30 歲時戒煙，更可賺回十年壽命。

Q: 成功戒煙必須依靠戒煙輔助藥物?

A: 當吸煙者開始戒煙，身體可能會因尼古丁水平減少而出現退癮徵狀。 研究指出，戒煙輔助藥物能紓緩退癮徵狀，有效提升戒煙成功率。 使用戒煙輔助藥物如戒煙貼、戒煙香口膠是戒煙方法的一種，但並非 必需，部分吸煙人士亦能以個人意志或其他方法戒掉煙癮。在使用任 何戒煙輔助藥物之前，應先諮詢醫護人員或戒煙輔導員。無論用什麼 戒煙方法，意志和恆心是必需的。

Q ： 戒煙後，有什麼需要注意?

A ： 戒煙人士應該建立健康的生活模式，包括均衡飲食、適量運動及維持 健康心理。擴闊生活圈子及跟親友傾訴亦很重要，讓自己在各方面的 生活得到平衡，必可完全戒掉煙癮。

Q: 戒煙令人體重增加?

A: 吸煙人士味覺受到煙害破壞，而戒煙後，身體的機能逐漸回復，味覺 和嗅覺都得到改善。假如不注意飲食，體重有所增加也不足為奇，但 只要保持均衡飲食及做適量運動，便可以保持健美。

Q: 戒煙後會否患上嚴重疾病如肺癌、心臟病?

A: 戒煙只會讓身體機能恢復。只要戒煙人士能保持堅定的意志，拒絕吸 煙，患上嚴重疾病的機會將會大大減低。有些長期吸煙人士在戒煙後 被診斷患上致命疾病，往往是因為長期吸煙，煙草已對健康造成嚴重 影響。想身體健康，戒煙是必要的。

Q ： 如果戒煙失敗，代表我再沒可能戒掉煙癮嗎?

A ： 你絕對能戒掉煙癮的! 只要檢討上次戒煙的失敗原因，避免犯下同樣 錯誤，重新設定戒煙計劃，如有需要，配合戒煙輔導員的指引及戒煙 輔助藥物，戒煙效果必會事半功倍。

5.尼古丁替代法

戒煙貼

戒煙貼的使用方法非常簡單，每天把一片戒煙貼片貼在清潔乾爽的皮膚上，在 游泳和洗澡期間也可以繼續使用，但必須每天更改附貼位置，以減低出現皮膚 敏感的機會。

戒煙香口膠

戒煙香口膠的咀嚼方法與一般香口膠不同，先把香口膠慢慢咀嚼 10 至 15 次， 然後置於口腔壁與牙肉之間讓尼古丁吸收；再次重複以上步驟，直到香口膠完 全無味道為止。

戒煙糖

戒煙糖有不同劑量可供選擇，使用戒煙糖時應把糖輪流放在口腔的兩邊直至完 全溶解，不可咬碎或整粒吞下。
