# Supplementary material for: Adaptive interventions to optimise the mobile phone-based smoking cessation support: study protocol for a sequential, multiple assignment, randomised trial (SMART)
Source: Trials. 2022 Aug 18;23:681. doi: 10.1186/s13063-022-06502-7 (PMC9387009; doi:10.1186/s13063-022-06502-7)
Supplement: Supplementary file 4 — Additional file 4: Appendix 4. Consent form. [file 13063_2022_6502_MOESM4_ESM.pdf]

COSH 香港吸煙與健康委員會 HONG KONG COUNCIL ON SMOKING AND HEALTH

香港大學
護理學院
公共衛生學院

第十屆「戒煙大贏家」無煙社區計劃

參加者自願參加同意書

香港大學護理學院及公共衛生學院受香港吸煙與健康委員會委託，現正進行一項為吸煙者提供即場輔導跟進及獎勵戒煙的「戒煙大贏家」比賽和隨機對照研究。如果你願意參與這項比賽和研究，請你填寫一份關於吸煙情況的問卷。我們會即場測量你的一氧化碳水平及提供戒煙輔導，整個輔導過程大約需時 5 分鐘。我們亦會提供有關戒煙的資料，包括介紹一些戒煙方法及吸煙的害處等。

如果你同意參與這項研究，你將會於第一、第二、第三及第六個月，接收到共 4 次電話跟進訪問和計劃的資訊，每次電話跟進過程大約需時 8 分鐘。若你成功完成所有跟進活動，則可以獲得現金獎港幣$100。此外，如你於第三及六個月跟進訪問時已經戒煙 7 天或以上，我們將邀請你進行一氧化碳及可的寧水平測量，每次核實成功戒煙可獲得港幣$500 獎金。核實測量內容：用一氧化碳測定儀量度你呼氣中的一氧化碳水平及使用可的寧測試紙測量你吐出的口水中的可的寧水平。

這項研究絕對安全，不會令你產生不安。根據香港法律《個人資料(私隱)條例》(第 486 章)，所有收集的資料會絕對保密，例如在本項研究中或與本項研究有關的個人資料的收集、保管、保留、管理、控制、使用(分析或比較)、在香港內外轉讓、不披露、消除和/或任何方式處理。另外，參加者須為其在電話通訊網路傳送的任何留言內容負上全部責任。輔導員有權刪除不適當言論、圖片及任何檔案。若參加者違反以上守則，會給予警告，嚴重者或會被取消其參與輔導服務的資格。此研究經已由香港大學及醫管局港島西醫院聯網研究倫理委員會審閱及批准。你可以選擇是否參與這項研究及有權隨時退出而不影響我們提供之服務。香港大學護理學院及公共衛生學院保留任何爭議的最終決定權。如果你希望知道你本人的測試結果或對整項研究結果或有任何疑問，請聯絡我們的研究團隊：

計劃總監：王文炳博士　　電話 3917-6636
計劃統籌：翁　雪博士　　電話 3917-6304　　劉正彥先生　　電話 3917-6951

（請在適當方格填上√）:

		是	否
1.	本人已閱畢及明白上述資料，及有機會對這項研究提出查詢。		
2.	本人**自願**參與是項研究，並知道有權隨時退出，不用作出任何解釋。這將不會影響本人所接受的服務或權益。		
3.	本人明白所有個人資料會完全保密，只有授權主要研究者及其研究團隊和香港大學及醫管局港島西醫院聯網研究倫理委員會獲得，並只會用於研究用途。		
4.	我**同意**參與這項研究，並同意研究員日後聯絡我，以便跟進。		

________________	________________	________________
被訪者姓名（請用正楷）	簽署	日期
________________	________________	________________
研究員姓名（請用正楷）	簽署	日期
王文炳博士	________________	________________
計劃總監姓名（請用正楷）	簽署	日期
